# Recommended Levels of Physical Activity Are Associated with Reduced Risk of the Metabolic Syndrome in Mexican-Americans

**DOI:** 10.1371/journal.pone.0152896

**Published:** 2016-04-07

**Authors:** Shenghui Wu, Susan P. Fisher-Hoch, Belinda Reininger, Joseph B. McCormick

**Affiliations:** 1 Department of Epidemiology & Biostatistics, University of Texas Health Science Center at San Antonio-Laredo Campus, Laredo, Texas, United States of America; 2 Division of Epidemiology, University of Texas Health Science Center-Houston, School of Public Health, Brownsville Campus, Brownsville, Texas, United States of America; 3 Division of Health Promotion and Health Behavior University of Texas Health Science Center-Houston, School of Public Health, Brownsville Campus, Brownsville, Texas, United States of America; Shanghai Institute of Hypertension, CHINA

## Abstract

**Purpose:**

To measure the association between physical activity and the metabolic syndrome risk in Mexican-Americans.

**Methods:**

Participants were drawn from the Cameron County Hispanic Cohort (n = 3,414), a randomly selected Mexican-American cohort in Texas on the US-Mexico border. Moderate and vigorous physical activity was assessed using reliable and validated instruments. The metabolic syndrome was defined as having 3 or more metabolic abnormalities.

**Results:**

One thousand five hundred and twenty-four participants of the cohort (45.02%) were found to have the metabolic syndrome. Compared to participants who did not meet US physical activity guidelines, participants who met physical activity guidelines of 150 moderate and vigorous minutes per week (≥ 600 MET adjusted minutes) had 36% lower risk for the metabolic syndrome (OR = 0.64; 95% CI: 0.42–0.98), and participants with total minutes per week of moderate and vigorous/strenuous activity greater than 743 MET adjusted minutes had 37% lower risk for the metabolic syndrome (OR = 0.63; 95% CI: 0.42–0.94) compared with their counterparts, after adjusting for age, gender, annual household income, body mass index, smoking and alcohol drinking status, total portions of fruit and vegetable intake, census tracts and blocks, and survey version for physical activity.

**Conclusions:**

Meeting or exceeding physical activity guidelines significantly was inversely associated with the risk for the metabolic syndrome in Mexican-Americans. Improving levels of physical activity appears to be an effective target for the metabolic syndrome prevention and control among Mexican-Americans independent of other factors.

## Introduction

The metabolic syndrome is a major public health problem worldwide[1]. The metabolic syndrome is characterized by a clustering of risk factors, including obesity, hypertension, dyslipidemia, hyperglycemia, and hyperinsulinemia [2,3]. The National Health and Nutrition Examination Survey reported that using the National Cholesterol Education Program definition of the metabolic syndrome [2,3], the age-adjusted prevalence of the metabolic syndrome was 44.5% among Hispanic men, and 44.1% among Hispanic women [4]. It is now widely accepted that the metabolic syndrome has an important mediating role in the increased risk of cardiovascular disease and type 2 diabetes[2,3,5]. Prevention of the metabolic syndrome and treatment of its main characteristics are now considered of utmost importance in order to combat the epidemic of type 2 diabetes mellitus and to reduce the increased risk of cardiovascular disease and all-cause mortality[6]. As a modifiable lifestyle factor, the role of physical activity in health has been increasingly recognized [7]. However, the potential importance of physical activity in the development of the metabolic abnormalities has been less extensively investigated compared with other lifestyle changes and factors [6]. One meta-analysis pooling prospective cohort studies evaluated the association between physical activity and risk of the metabolic syndrome[8]. This meta-analysis of 17 cohort studies reported that a high level of leisure time physical activity was statistically associated with decreased risk of the metabolic syndrome [high vs. low: relative risk (RR) = 0.80, 95% confidence interval (CI) 0.75–0.85], whereas a moderate level of leisure time physical activity was weakly associated with decreased risk of the metabolic syndrome (moderate vs. low: RR = 0.95, 95% CI 0.91–1.00) [8]. However, compared with low level leisure time physical activity, Americans with high level leisure time physical activity did not significantly reduce the risk of the metabolic syndrome (all 95% CIs for each individual study across 1) based on four studies from the USA included into the meta-analysis [8]. Only one study conducted in Mexicans [9] reported the reduced risk of the metabolic syndrome from physical activity in Mexico, but not in Mexican-Americans in the US. The objective of this study was to explore the effect of physical activity on metabolic syndrome risk in a randomly selected cohort of Mexican-American subjects.

## Materials and Methods

### Study Participants

This study was approved by the Committee for the Protection of Human Subjects of the UT Health, Houston and the Institutional Review Board of the University of the Texas Health Science Center, San Antonio. All study participants gave written informed consent. This cross-sectional analysis used data from the Cameron County Hispanic Cohort (CCHC), an ongoing homogenous community-dwelling Mexican-American cohort study [10,11]. Study subjects were recruited from randomly selected tract/blocks according to the 2000 Census as described previously [10,11]. At the baseline survey conducted between 2003 and 2015, 3,414 participants aged 18 years or older were recruited from their households in three predominantly Mexican-American cities along the Rio Grande Border with Mexico. All participants recruited between 2003 and 2012 were from Brownsville, TX.

All subjects responded to a detailed baseline survey of demographic characteristics, lifestyle including diet, physical activity, family and medical history, and other exposures. Participants were asked to fast for at least 10 hours overnight before a clinic visit at the Clinical Research Unit. Anthropometric measurements, including current weight, height, and circumferences of the waist and hip, were also taken [10,11]. Physical activity in a typical week according to intensity, frequency (times / week) and duration (minutes / time) was assessed using the International Physical Activity Questionnaire short-form (IPAQ)[12] or the Godin Leisure-Time Exercise Questionnaire instruments [13] as reported previously [14]. The instruments have reported evidence of reliability and validity [12,15–18]. Physical activity energy expenditure was estimated using standard metabolic equivalent (MET) values [19]. A MET is the ratio of the rate of energy expended during an activity to the rate of energy expended at rest. For example, 1 MET is the rate of energy expenditure while at rest. A 4 MET activity expends 4 times the energy used by the body at rest. If a person does a 4 MET activity for 30 minutes, he or she has done 4 × 30 = 120 MET-minutes (or 2.0 MET-hours) of physical activity [19]. Moderate-intensity activities are defined as 3.0 to 5.9 METs. Walking at 3.0 miles per hour requires 3.3 METs of energy expenditure and is therefore considered a moderate-intensity activity. Vigorous-intensity activities are defined as 6.0 METs or more. Running at 10 minutes per mile (6.0 mph) is a 10 MET activity and is therefore classified as vigorous intensity [19]. In this study, the MET adjusted minutes of moderate and vigorous physical activity in the last week was calculated based on responses [11]. Physical activity ≥ 600 MET adjusted minutes was considered meeting United States physical activity guidelines (USDHHS, 2008)[19].

All participants responded to a detailed baseline survey that collected information on demographic characteristics, lifestyle and dietary histories, medical history, and other exposures. Using the Food Frequency Questionnaire[20], fruit and vegetable consumption was assessed by asking participants how many portions of fruit and vegetables they ate daily. A portion size was described as a ½ cup of fresh, frozen, or canned produce or a medium-sized piece of produce (such as 130–189 berries per cup) [21,22]. Consumption of five or more fruit and vegetable portions was considered meeting US guidelines[21,22]. Weight was measured to the nearest tenth of a kilogram and height to the nearest tenth of a centimeter. Body mass index (BMI) was calculated as weight in kilograms divided by height squared in meters (kg/m^2^). Waist circumference (WC) was measured at the level of the umbilicus and hip circumference (HC) at the level of maximum width of the buttocks with participants in a standing position and breathing normally, to the nearest 0.2 cm. Waist-to-hip ratio (WHR) was calculated as WC divided by HC [10]. The average of 3 blood pressures (BP) taken 5 minutes apart were used.

### Laboratory Measurements

All participants provided a blood sample at baseline. After collection, samples were placed on ice and centrifuged within 30 minutes of collection. Following processing and aliquoting, all samples were stored at -80°C until laboratory analyses were conducted. Laboratory studies performed included fasting lipid panel, hemoglobin (Hb) A1c and fasting plasma glucose was performed by a local CLIA certified laboratory. Fasting serum insulin was consistently performed in-house using Mercodia immunoassays (Uppsala, Sweden). Homeostasis model assessment insulin resistance index (HOMA-IR) was calculated as fasting glucose (mg/dL)/18 × fasting insulin (mU/L)/22.5 [23].

### Definition of the metabolic syndrome

The metabolic syndrome was defined as having ≥ 3 of the following metabolic abnormalities: waist circumference (WC) ≥ 102 cm in men or ≥ 88 cm in women; systolic BP (SBP) ≥ 130 mmHg and/or diastolic BP (DBP) ≥ 85 mmHg or on antihypertensive medication; triglyceride ≥ 150 mg/dL; high-density lipoprotein cholesterol < 40 mg/dL in men or < 50 mg/dL in women; fasting glucose ≥ 100 mg/dL or on diabetes medication [2,3].

### Statistical analysis

Descriptive results and the models reported in this paper were adjusted for the probability sampling weights also taking into consideration clustering effects arising from the census block and household [10]. Log-transformation was conducted to normalize the distribution of continuous variables as appropriate. Survey-weighted linear regression was used to obtain the t-test statistics to compare phenotypes and to be used for multiple pairwise mean comparisons for continuous data. Survey-weighted chi-square test was used to obtain Rao-Scott F adjusted chi-square statistic to compare phenotypes for categorical data. Survey-weighted logistic regression modeling was performed to estimate the ORs for metabolic syndrome risk and their 95% CIs by physical activity adjusting for other covariates. Potential confounders adjusted for in multivariable survey-weighted logistic regression models included age, gender, annual household income, BMI, alcohol drinking, cigarette smoking status, and discrepancies in survey versions for physical activity data collection. Physical activity was classified into two groups respectively according to the mean of the total minutes per week of moderate and vigorous/strenuous activity (743 MET adjusted minutes), and if participants met physical activity guidelines of 150 moderate and vigorous minutes per week (≥ 600 MET adjusted minutes). The unit of physical activity was changed from minutes to hours when it was continuous variable so as to avoid too small ORs.

To illustrate the dose-response relationship between physical activity and the metabolic syndrome, we also used a restricted cubic spline logistic regression analysis[24] to evaluate the risk of the metabolic syndrome with total MET adjusted hours per week of moderate and vigorous/strenuous activity. Knots were placed at the 5^th^, 50^th^, and 95^th^ percentiles of the distribution of age at enrollment.

Statistical analyses were carried out by using SAS version 9.3 (SAS Institute, Cary, NC). All statistical tests were based on 2-sided probability.

## Results

At the time of this study a total of 3,414 individuals were enrolled in the CCHC, 2,944 from Brownsville, 272 participants from Harlingen and 198 participants from Laredo, Texas. Based on the recommended scoring protocols, 29 (0.8%) participants with extreme values (≥ 7,680 MET adjusted minutes) of physical activity were excluded from the analyses. Of the remaining 3,385 participants, mean age of this subset was 45 years; 34% were male. A total of 12.6% (n = 427) of the participants met minimum recommendations for physical activity of ≥ 600 MET adjusted minutes per week.

One thousand five hundred and twenty-four participants of the cohort (45.02%) were found to have the metabolic syndrome and 1,861 did not report the metabolic syndrome (**Table 1**). Participants with the metabolic syndrome were more likely to be older, and less likely to meet the recommended guidelines for physical activity of more than 600 MET adjusted minutes per week or meet the recommended guidelines for fruit and vegetables more than 5 servings per day. They had lower all physical activity, moderate and vigorous activity and average daily time doing heavy activity, but higher BMI, WC, WHR, systolic and diastolic blood pressure, insulin, fasting blood glucose, HOMA-IR and HbA1c than their counterparts (all *P*s < 0.05). They showed significantly elevated mean values of triglycerides compared with subjects without the metabolic syndrome, but decreased values of high density lipid cholesterol (HDLC). Although levels of total cholesterol were higher and consumption of total portions of fruit and vegetables were lower in subjects with the metabolic syndrome than those without, the differences were not statistically significant. There was no difference in gender, annual household income, employed status, education level, current and ever cigarette smoking status, ever alcohol drinking, time spent in doing mild physical activity, and mean levels of low-density lipoprotein between subjects with and without the metabolic syndrome.

**Table 1:** Cohort Demographics and Metabolic Characteristics Stratified by Metabolic syndrome Risk: Cameron County Hispanic Cohort Study (2003–2015)^a^^,^^b^.

		Metabolic syndrome	
Variable	Yes (n = 1,524, 45.02%)	No (n = 1,861, 54.98%)	*P*-value
**Categorical variables, n (%)**			
Men	493 (32.35)	651 (34.98)	0.87
Employed	681 (44.69)	963 (51.75)	0.10
Education, below high school	875 (57.41)	853 (45.84)	0.71
Met minimum recommendations for physical activity of ≥ 600 MET-minutes/week	148 (9.71)	267 (14.35)	0.02
Met recommendations of ≥ 5 servings of fruit & vegetables per day	54 (3.54)	92 (4.94)	0.01
Current smokers	232 (15.22)	272 (14.62)	0.97
Ever smokers	474 (31.10)	535 (28.75)	0.84
Ever alcohol drinkers	807 (52.95)	917 (49.27)	0.71
**Continuous variables, Mean (standard error)**			
Age at enrollment (years)	51.75 (1.51)	43.80 (1.75)	0.001
Annual household income (US dollars)	38324 (6928)	63673 (32950)	0.45
MET minutes/wk. of all activity	667.54 (159.56)	1177.33 (181.07)	0.04
MET minutes/wk. of moderate and vigorous activity	463.75 (63.57)	1143.77 (130.36)	< .0001
MET adjusted minutes of mild activity	258.77 (59.74)	303.89 (75.35)	0.65
Average hours per day doing heavy activity	0.81 (0.08)	1.27 (0.10)	0.0003
Total portions of fruit and vegetables	2.19 (0.17)	2.46 (0.21)	0.31
Body mass index (kg/m^2^)	33.56 (0.57)	29.84 (0.58)	< .0001
Waist circumference (cm)	110.62 (1.30)	100.31 (1.47)	< .0001
Waist-to-hip ratio	0.97 (0.01)	0.92 (0.01)	< .0001
Total cholesterol (mg/dL)	188.01 (4.68)	181.87 (3.53)	0.28
Triglycerides (mg/dL)	187.31 (10.11)	108.65 (3.87)	< .0001
HDL-cholesterol (mg/dL)	43.19 (0.92)	53.14 (0.91)	< .0001
LDL-cholesterol (mg/dL)	108.05 (3.62)	108.11 (2.86)	0.99
High sensitivity C-reactive protein (mg/L)^c^	5.44 (1.05)	3.39 (1.09)	< .0001
Systolic blood pressure (mmHg)	132.14 (1.98)	119.25 (2.14)	< .0001
Diastolic blood pressure (mmHg)	79.24 (0.67)	74.70 (0.73)	< .0001
Insulin (mg/dL)^c^	15.74 (1.02)	9.86 (1.03)	< .0001
Fasting blood glucose (mg/dL) ^c^	118.76 (1.03)	94.47 (1.02)	< .0001
HOMA-IR^c^	4.68 (1.03)	2.34 (1.03)	< .0001
HbA1c (%)^c^	6.48 (1.02)	5.69 (1.01)	< .0001

^a^ Abbreviation: LDL: low-density lipoprotein; Hb: hemoglobin; HDL: high-density lipoprotein; HOMA IR: homeostatic model assessment insulin resistance; MET: metabolic equivalent

^b^ All descriptive results and the models were adjusted for the probability of sampling using weights taking into consideration clustering effects arising from the same census block and household. Linear regression models were used for continuous variables and Rao-Scott F adjusted chi-square statistic for categorical variables.

^c^ Geometric concentrations.

Participants who met physical activity guidelines of 150 moderate and vigorous minutes per week (≥ 600 MET adjusted minutes) had 31% lower risk for the metabolic syndrome (OR = 0.69; 95% CI: 0.54–0.88) after adjusting for age and gender compared with those who did not meet guidelines (**Table 2**). Multivariable-adjusted model showed that meeting physical activity guidelines was associated with 36% lower total risk for the metabolic syndrome (OR = 0.64; 95% CI: 0.42–0.98) after further adjusting for annual household income, smoking, alcohol drinking, BMI, total consumption of fruit and vegetables, census tracts and blocks, and discrepancies in survey versions for physical activity data collection. Compared to participants with total minutes per week of moderate and vigorous/strenuous activity less than 743 MET adjusted minutes, participants with those greater than 743 MET adjusted minutes had 33% (OR = 0.67; 95% CI: 0.52–0.86) lower risk of the metabolic syndrome after adjusting for age and gender (**Table 2**). Multivariable-adjusted model showed that participants with those greater than 743 MET adjusted minutes had 37% lower risk (OR = 0.63; 95% CI: 0.42–0.94).

**Table 2:** Association between physical activity and risk for the metabolic syndrome.

Physical activity	Age, gender-adjusted model		Multivariable-adjusted model^a^	
	OR (95% CI)	*P*	OR (95% CI)	*P*
Meet physical activity guidelines of 150 moderate and vigorous minutes per week				
<600 metabolic equivalents	Reference		Reference	
≥600 metabolic equivalents	0.69 (0.54–0.88)	0.003	0.64 (0.42–0.98)	0.04
Total minutes per week of moderate and vigorous/strenuous activity^b^				
<743 metabolic equivalents	Reference		Reference	
≥743 metabolic equivalents	0.67 (0.52–0.86)	0.002	0.63 (0.42–0.94)	0.02

OR: odds ratio; CI: confidence interval; Ref: reference

^a^ Multivariable adjusted model: adjusted for age, gender, annual household income, smoking, alcohol drinking, body mass index, total consumption of fruit and vegetables, census tracts and blocks, and discrepancies in survey versions for physical activity data collection.

^b^ Two groups were classified according to the median of the total minutes per week of moderate and vigorous/strenuous activity (743 metabolic equivalents).

**Fig 1:**
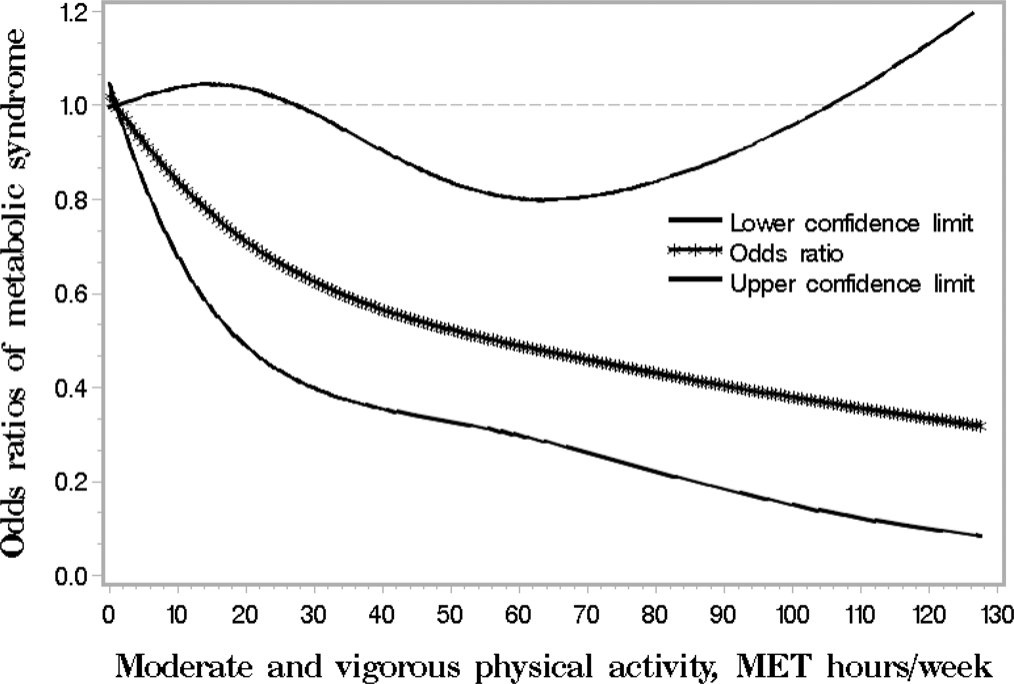
Smoothed plot for odds ratios (ORs) of the metabolic syndrome risk according to total metabolic equivalent (MET) hours of physical activity per week. The ORs were estimated by using the restricted cubic-spline logistic regression models with knots placed at the 5^th^, 50^th^, and 95^th^ percentiles of physical activity. The overall association between physical activity and the risk of the metabolic syndrome was significant (*P* = 0.02). The model was adjusted for age, sex, annual household income, cigarette smoking status, alcohol drinking status, body mass index, total consumption of fruit and vegetables, discrepancies in survey versions for physical activity data collection and the probability of sampling using weights taking into consideration clustering effects arising from the same census block and household.

**Fig 1**visually depicts the shape of the dose-response relationship between physical activity and the metabolic syndrome risk after adjusting for potential confounding variables in a restricted cubic spline model. Total metabolic equivalent hours of physical activity per week was inversely associated with the risk for the metabolic syndrome (*P* for overall association = 0.02). The linear trend was not statistically significant (*P* = 0.12). Although ORs less than 30 MET hours/week were not statistically significant (95% CIs across 1) in **Fig 1**, the findings were not conflicted with **Table 2**due to the different purposes and meanings of ORs in **Fig 1**and **Table 2**. ORs in **Table 2**were comparisons between participants with MET adjusted minutes ≥ 600 and those with MET adjusted minutes < 600 or between participants with MET adjusted minutes ≥ 743 and those with MET adjusted minutes < 743. ORs in **Fig 1**were the odds ratios of the metabolic syndrome corresponding to MET adjusted hours per week in moderate and vigorous activity.

## Discussion

In a Mexican-American cohort, participants who met physical activity guidelines of 150 moderate and vigorous minutes per week (≥ 600 MET adjusted minutes) had significantly 36% lower for the metabolic syndrome risk, and participants with total minutes per week of moderate and vigorous/strenuous activity greater than 743 MET adjusted minutes had significantly 37% lower for the metabolic syndrome risk, compared with their corresponding counterparts, after adjusting for covariates. Cubic spline logistic regression showed the dose-response relationship that total metabolic equivalent hours of moderate and vigorous physical activity per week was inversely associated with the risk for the metabolic syndrome.

To our knowledge, this study is the first to examine the inverse association between moderate and vigorous physical activity and the risk for the metabolic syndrome among Mexican-Americans. According to the United States physical activity guidelines (USDHHS, 2008)[19], moderate vigorous physical activities in this study include walking briskly (3 miles per hour or faster, but not race-walking), water aerobics, bicycling slower than 10 miles per hour, tennis (doubles), ballroom dancing and general gardening; vigorous physical activities include race walking, jogging, or running, swimming laps, tennis (singles), aerobic dancing, bicycling 10 miles per hour or faster, jumping rope, heavy gardening (continuous digging or hoeing, with heart rate increases) and hiking uphill or with a heavy backpack. Prior cross-sectional studies found an inverse gradient between amount of physical activity and the metabolic syndrome in participants with mixed ethnicities adjusting for ethnicity and other covariates [25,26]. One meta-analyses of prospective cohort studies conducted in people from Europe, America, Asia and South America reported that a high level of leisure time physical activity was statistically associated with decreased risk of the metabolic syndrome[8], however, a moderate level of leisure time physical activity was non-significantly associated with decreased risk of the metabolic syndrome [8], and Americans with high level leisure time physical activity did not statistically significantly reduce the risk of the metabolic syndrome based on the four studies from USA included into the meta-analysis [8]. Only one study conducted in Mexico [9] showed that the metabolic syndrome risk was reduced among men (OR = 0.72; 95% CI = 0.57–0.95) and women (OR = 0.78; 95% CI = 0.64–0.94) who reported an amount of ≥ 30 minutes/day of leisure time activity, and among women who reported an amount of ≥ 3 hours/day of workplace activity (OR = 0.75; 95% CI = 0.59–0.96)[9]. This study further provided evidence showing the beneficial effect of physical activity on the risk for the metabolic syndrome in Mexican-Americans. Compared to participants who did not meet US physical activity guidelines, participants who met physical activity guidelines of 150 moderate and vigorous minutes per week (≥ 600 MET adjusted minutes) had 36% lower risk for the metabolic syndrome. This study is also the first to report the inverse dose-response relationship between moderate and vigorous physical activity and the risk for the metabolic syndrome. We found the dose-response relationship is also shown in the cubic spline regression (**Fig 1**). This study suggested that even 1 hour moderate and vigorous physical activity per week is helpful to lower the risk of the metabolic syndrome. Although further longitudinal data is needed, our findings still provide important information for the prevention and control of the metabolic syndrome and related conditions among Mexican-Americans.

Potential mechanisms influenced by exercise involved in the components of the metabolic syndrome may include alterations in abdominal adipose tissue accumulation[27], glucose [27,28], dyslipidaemia[6] and blood pressure [29]. Genetic predisposition (related to several candidate genes[30]) in combination with the effect of environmental factors is likely to influence the development of insulin resistance and/or the clinical expression of the metabolic syndrome[6]. Consistently, we found that participants with physical activity levels < 743 MET adjusted minutes had higher levels of WC (106.4 vs. 101.4; *P* = 0.01), systolic blood pressure (125.4 vs 118.2 mmHg; *P* = 0.01), diastolic blood pressure (76.2 vs 74.8 mmHg; *P* = 0.20), fasting blood glucose (113.6 vs. 97.1 mg/dL; *P* < 0.0001) and triglyceride (161.2 vs. 136.5 mg/dL; *P* = 0.01), but lower levels of HDLC (45.59 vs 50.74 mg/dL; *P* = 0.048), compared to participants with physical activity levels ≥ 743 MET adjusted minutes.

There are some methodological limitations in our research. The study was cross-sectional in design; thus, only associations but not causal relationship may be inferred. Prospective studies are needed to further investigate the effect or dose-response of physical activity on metabolic syndrome risk. Our longitudinal data currently being collected will provide that opportunity. Physical activity was self-reported, which may affect its precision as a predictor. We could not completely rule out the possibility of residual confounding due to unmeasured or inadequately measured covariates.

This study had several strengths. First, this is a general population-based randomly selected Mexican-American cohort with relatively large sample size, thus avoiding bias inherent in studies drawing from clinic populations or other non-randomly selected populations with established disease or mixed ethnicity. Second, we first found an inverse dose-response relationship between physical activity and the metabolic syndrome in Mexican-Americans. Finally, detailed assessment of physical activity and information on a wide range of factors related to the metabolic syndrome was available, allowing us to get a relatively comprehensive analysis of the relevant factors. If having the metabolic syndrome might have increased physical activity as a result we did not observe this in our robust baseline cohort population.

## Conclusion

Increased moderate and vigorous physical activity was associated with a significant risk for the metabolic syndrome after excluding the effect of other confounding factors. Therefore, physical activity might be a modifiable protective factor for which Mexican-Americans can make changes to reduce their metabolic syndrome risk. Efforts need to be focused on improving physical activity intervention.

## Abbreviations

BMI: Body mass index
BP: blood pressures
CCHC: Cameron County Hispanic Cohort
CI: confidence interval
CRP: C-reactive protein
DBP: diastolic blood pressures
Hb: hemoglobin
HC: hip circumference
HDLC: high density lipid cholesterol
HOMA-IR: Homeostasis model assessment insulin resistance index
IPAQ: International Physical Activity Questionnaire short-form
LDL: low-density lipoprotein
MET: metabolic equivalent
OR: odds ratio
RR: relative risk
SBP: systolic blood pressures
WC: waist circumference
WHR: waist-to-hip ratio
